# Doctors in Atlanta

**Published:** 1885-10-20

**Authors:** 


DOCTORS IN ATLANTA.—We had a call from Mr. D. R. Bogue, the genial and polite agent of R. L. Polk & Co., who are now getting out the New Medical and Surgical Directory of the United States. It will be a great and useful work to the profession, and to all who have use for the names of medical men of the United States. It will contain also other highly useful information. Mr. Bogue has thoroughly canvassed the city of Atlanta in the search for information for the Directory. In conversation with him, we gathered the following facts as to Atlanta doctors. There are in Atlanta a total of 155 Doctors, as follows:

| | |
|---|---|
| Regulars | 110 |
| Hygeo-Therapeutists | 2 |
| Homœpaths | 11 |
| Eclectics | 31 |
| Unknown | 1 |

There are six female physicians included in the above, and two colored doctors.

---

DEATH OF WILLIAM BRAITHWAITE, M. D.—We learn by a note from Dr. W. A. Townsend that mail advices from England announce the death of the well known English physician and surgeon, William Braithwaite, the founder of The Retrospect of Medicine, who died at his home in Leeds on January 31. The Yorkshire Post of February 2 says: "He was the oldest medical practitioner in Leeds, and in his large and varied practice he was esteemed on all hands, both on account of his great knowledge and his sympathetic and kindly disposition. Dr. Braithwaite was born in 1807, and was, therefore, in his seventy-eighth year."

A letter received by Dr. Townsend, dated February 8, from Dr. James Braithwaite, says: "I grieve to inform you of my father's death, which occurred on January 31 last. He died without any suffering and from failure of the heart, which had been noticeable for twelve months previously. I shall carry on The Retrospect with the assistance of Dr. A. G. Barre, assistant physician to the Leeds General Infirmary. I have done all the heavy work on the book for twenty-five years."

---

MATERIA MEDICA COLLECTION FOR STUDENTS OF PHARMACY AND MEDICINE, containing specimens of all Crude Drugs of Vegetable Origin recognized in the U. S. Pharmacopeia. and many not so recognized that are in common use—in all 288 specimens—indispensable to the Student of Pharmacognosy; put up in a beautiful case (length of case, 23 inches; width, 16 inches; depth, 13½ inches), by Parke, Davis & Co., Detroit, Mich.

The manufacturers truly state that "The student can familiarize himself, practically, with the properties of drugs only as he has the opportunity to examine and handle specimens himself. . . . Specimens of many of the common drugs are, of course, easily procured at any drug store for such examinations, but there are many which are not thus accessible, and it is, moreover, important that the specimens shall be all of unquestionable authenticity. The present collection furnishes this desideratum."

This furnishes another evidence of the remarkable energy and enterprise of the house of Parke, Davis & Co. They are entitled to the thanks of the Profession, and may justly be numbered among the highest of those who, by their skill and enterprise, have brought honor and distinction upon the American people.

